# Crucial Role for Basophils in Acquired Protective Immunity to Tick Infestation

**DOI:** 10.3389/fphys.2018.01769

**Published:** 2018-12-07

**Authors:** Hajime Karasuyama, Yuya Tabakawa, Takuya Ohta, Takeshi Wada, Soichiro Yoshikawa

**Affiliations:** ^1^Department of Immune Regulation, Graduate School of Medical and Dental Sciences, Tokyo Medical and Dental University, Tokyo, Japan; ^2^Division of Molecular Medicine, Institute of Advanced Medical Sciences, Tokushima University, Tokushima, Japan

**Keywords:** basophil, mast cell, tick resistance, IgE, histamine

## Abstract

Ticks are blood-sucking arthropods that can transmit various pathogenic organisms to host animals and humans, causing serious infectious diseases including Lyme disease. Tick feeding induces innate and acquired immune responses in host animals, depending on the combination of different species of animals and ticks. Acquired tick resistance (ATR) can diminish the chance of pathogen transmission from infected ticks to the host. Hence, the elucidation of cellular and molecular mechanism underlying ATR is important for the development of efficient anti-tick vaccines. In this review article, we briefly overview the history of studies on ATR and summarize recent findings, particularly focusing on the role for basophils in the manifestation of ATR. In several animal species, including cattle, guinea pigs, rabbits and mice, basophil accumulation is observed at the tick re-infestation site, even though the frequency of basophils among cellular infiltrates varies in different animal species, ranging from approximately 3% in mice to 70% in guinea pigs. Skin-resident, memory CD4^+^ T cells contribute to the recruitment of basophils to the tick re-infestation site through production of IL-3 in mice. Depletion of basophils before the tick re-infestation abolishes ATR in guinea pigs infested with *Amblyomma americanum* and mice infested with *Haemaphysalis longicornis*, demonstrating the crucial role of basophils in the manifestation of ATR. The activation of basophils via IgE and its receptor FcεRI is essential for ATR in mice. Histamine released from activated basophils functions as an important effector molecule in murine ATR, probably through promotion of epidermal hyperplasia which interferes with tick attachment or blood feeding in the skin. Accumulating evidence suggests the following scenario. The 1^st^ tick infestation triggers the production of IgE against tick saliva antigens in the host, and blood-circulating basophils bind such IgE on the cell surface via FcεRI. In the 2^nd^ infestation, IgE-armed basophils are recruited to tick-feeding sites and stimulated by tick saliva antigens to release histamine that promotes epidermal hyperplasia, contributing to ATR. Further studies are needed to clarify whether this scenario in mice can be applied to ATR in other animal species and humans.

## Introduction

Ticks, particularly ixodid family members, are blood-sucking ectoparasites of vertebrates and can transmit various pathogens to animals and humans during blood feeding for days, causing serious infectious diseases, including Lyme disease, babesiosis, Rocky Mountain spotted fever, human monocytic ehrlichiosis and severe fever with thrombocytopenia syndrome (Gratz, 1999; Parola and Raoult, 2001; de la Fuente et al., 2008; Embers and Narasimhan, 2013; Wikel, 2013; Yamaji et al., 2018). Besides tick-borne infectious diseases, some people with the experience of tick bites show recurrent episodes of anaphylaxis, a life-threatening systemic allergic reaction, after eating red meat or treating with anticancer monoclonal antibodies (Platts-Mills and Commins, 2013; Steinke et al., 2015). Thus, tick infestation is of medical and veterinary public health importance.

Host defense mechanism is a threat to successful blood feeding by ticks and hence must be counteracted. To this end, ticks inject saliva containing various bioactive substances into the host during tick infestation, including vasodilator and antihemostatic, antiinflammatory and immunosuppressive reagents (Wikel, 2013). On the other hand, some animals, such as mice, guinea pigs, rabbits and cattle, have been shown to develop the resistance to tick feeding after single or multiple tick infestations, depending on the combination of animal species/strains and tick species (Trager, 1939; Wikel, 1996). This acquired tick resistance (ATR) is commonly assessed by several parameters, including the reduction in the number and/or body weight of engorged ticks or tick death when sensitized animals are re-infested with ticks. ATR was first described in 1938 by Trager who found that after infestation with *Dermacentor variabilis*, guinea pigs develop resistance to subsequent tick infestations (Trager, 1939). Since then, ATR has been further characterized by using cattle and laboratory animals including guinea pigs (Wikel, 1996). ATR is not restricted to the skin lesion of previous tick bites and can be observed in un-infested skin of sensitized animals, indicating the contribution of systemic responses rather than a localized response at the previously infested skin lesion. Moreover, ATR can be transferred to naive animals with sera or cells isolated from previously infested animals (Wikel and Allen, 1976; Brown and Askenase, 1981; Askenase et al., 1982), suggesting that ATR is a type of immune reaction. Importantly, ATR can diminish the chance of pathogen transmission from infected ticks to host animals and humans (Bell et al., 1979; Wikel et al., 1997; Nazario et al., 1998; Burke et al., 2005; Dai et al., 2009). Therefore, the elucidation of cellular and molecular mechanisms underlying ATR is important for developing efficient anti-tick vaccines that can minimize the transmission of pathogens causing serious infectious diseases.

Basophils are the least abundant granulocytes and account for less than 1% of peripheral blood leukocytes (Galli, 2000). They are named after basophilic granules in the cytoplasm that stain with basic dye, as first documented by Paul Ehrlich in 1879. In addition to basophilic granules, blood-circulating basophils share some phenotypic properties with tissue-resident mast cells, such as the surface expression of the high-affinity IgE receptor FcεRI and the release of allergy-inducing chemical mediators, including histamine, in response to various stimuli (Galli, 2000; Stone et al., 2010). Therefore, basophils have often been erroneously considered as minor and redundant relatives or blood-circulating precursors of tissue-resident mast cells (Falcone et al., 2000). It is now accepted well that basophils and mast cells are distinct cell lineages, and that basophils play crucial and non-redundant roles distinct from those played by mast cells (Voehringer, 2017; Karasuyama et al., 2018; Varricchi et al., 2018). Basophils contribute to protective immunity, particularly to parasitic infections while they are involved in the pathogenesis of various disorders, including allergic and autoimmune disorders.

In this review article, we summarize recent advances in our understanding of the cellular and molecular mechanisms underlying ATR, particularly focusing on the role of basophils identified mainly in mouse models of tick infestation.

## Basophils are Key Effector Cells in the Manifestation of ATR

### In Guinea Pigs

An early study described that cutaneous reactions at tick-feeding sites in tick-resistant guinea pigs were characterized by granulocytic inflammatory infiltrates, edema, and epidermal hyperplasia whereas the 1^st^ tick-feeding site in previously uninfested guinea pigs showed minimal skin reactivity (Trager, 1939). Accumulation of numerous basophils and eosinophils, with basophils comprising up to 70% of cellular infiltrates, was detected at tick-feeding sites of guinea pigs that manifested ATR (Allen, 1973). Such basophil-rich cutaneous reaction was referred as cutaneous basophil hypersensitivity (CBH) and extensively studied in 1970s and early 1980s (Katz, 1978). Basophil depletion in *A. americanum*-infested guinea pigs by using antiserum raised against basophils abolished ATR (Brown et al., 1982), demonstrating the important role for basophils in ATR. Basophil infiltration at the site of tick re-infestation was also observed in cattle and rabbits (Allen et al., 1977; Brossard and Fivaz, 1982), even though the frequency of basophils among cellular infiltrates varied, and the functional role of basophils in these animals has not yet been determined to our knowledge. Thus, it remained elusive whether the important finding on basophils in guinea pig ATR can be generalized to other animal species and humans.

### In Mice

A previous study reported that basophil infiltration was hardly detected at the tick-feeding site of WBB6F1-+/+ mice during re-infestation with *H. longicornis*, in spite of the fact that the mice showed ATR (Matsuda et al., 1990). Mast cell-deficient WBB6F1-*W/W^v^* mice failed to manifest ATR, and adoptive transfer of mast cells conferred ATR on these mice (Matsuda et al., 1985, 1987, 1990), suggesting that mast cells in place of basophils contributed to ATR in mice, unlike in guinea pigs. On the contrary, other studies reported that the same mast cell-deficient strain of mice showed ATR to another tick species *Dermacentor variabilis* (denHollander and Allen, 1985; Steeves and Allen, 1991). Murine basophils had been notoriously difficult to identify owing to their fewer basophilic granules compared to those in other animals and humans, and therefore, electron microscopic examination was needed to identify them in tissue sections (Urbina et al., 1981; Dvorak et al., 1982; Dvorak, 2000). Notably, the infiltration of basophils, along with eosinophils and neutrophils, was detected by electron microscopy at the tick-feeding site in the 3^rd^ infestation with *D. variabilis* in both mast cell-sufficient and -deficient mice (Steeves and Allen, 1991). Thus, the mechanism underlying ATR in mice, including the distinct roles played by basophils and mast cells, and the influence of different genetic background of both mice and ticks remained to be clarified.

**FIGURE 1:**
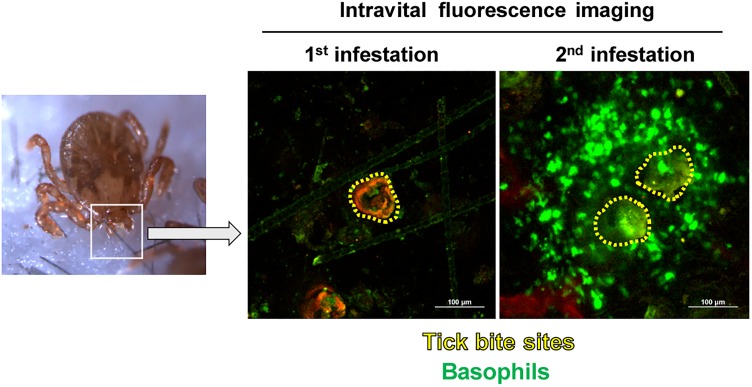
Basophils accumulate at the tick-feeding site during the 2^nd^ but not 1^st^ tick infestation. *Mcpt8*^GFP^ (green basophil) mice were infested with ticks one or twice and subjected to intravital fluorescence imaging analysis of green basophils at tick-feeding sites on day 2 of the 1^st^ or 2^nd^ infestation.

Recent characterization of cell surface markers on murine basophils (Min et al., 2004; Voehringer et al., 2004) and the identification of murine basophil-specific serine protease, mouse mast cell protease-8 (mMCP-8) (Poorafshar et al., 2000; Ugajin et al., 2009) have enabled us to identify and isolate murine basophils much more easily. Taking the advantage of a mMCP-8-specific mAb TUG8 (Ugajin et al., 2009), we demonstrated that mMCP-8-expressing basophils are recruited to the tick-feeding site and make a cluster around the tick mouthpart during the 2^nd^ but rarely the 1^st^ infestation with *H. longicornis* in C57BL/6 mice (Wada et al., 2010). Intravital fluorescence microscopic analysis, using *Mcpt8*^GFP^ (green basophil) mice in that only basophils express green fluorescent protein (GFP), confirmed the basophil accumulation at the 2^nd^ but not 1^st^ tick-feeding site (Ohta et al., 2017; Figure 1). Basophils represented less than 5% of leukocytes at the 2^nd^ tick-feeding site in mice, much fewer than in guinea pigs, while monocytes/macrophages, neutrophils and eosinophils were abundant. Importantly, we found that basophil depletion by treating mice with basophil-depleting mAbs, either anti-FcεRIα (MAR-1) or anti-CD200R3 (Ba103), just before the 2^nd^ tick infestation completely abolished ATR with no apparent effect on the number of other types of cells, including monocytes/macrophages, neutrophils and eosinophils (Wada et al., 2010). The essential role of basophils in ATR was further demonstrated by diphtheria toxin-mediated ablation of basophils in genetically engineered *Mcpt8*^DTR^ mice in that only basophils expressed diphtheria toxin receptors (Wada et al., 2010). Of note, we also demonstrated that mast cell-deficient *Kit^W-sh/W-sh^* C57BL/6 mice failed to manifest ATR, confirming the importance of mast cells in ATR reported previously (Matsuda et al., 1985, 1987, 1990). Thus, mast cells, in addition to basophils, appear to contribute to ATR in C57BL/6 mice infested with *H. longicornis*, in contrast to ATR in *D. variabilis*-infested WBB6F1-+/+ mice in that mast cells are dispensable (denHollander and Allen, 1985; Steeves and Allen, 1991).

### In Humans

Basophil infiltration was detected in humans at the tick-feeding sites and in the skin lesions of scabies (Ito et al., 2011; Nakahigashi et al., 2013; Kimura et al., 2017). Of note, a patient lacking basophils and eosinophils reportedly suffered from widespread scabies (Juhlin and Michaelsson, 1977). These observations suggest the possible involvement of basophils in protective immunity to ectoparasites, including ticks.

## Basophil Activation Through IgE and Its Receptor FcεRI is Essential for ATR

**FIGURE 2:**
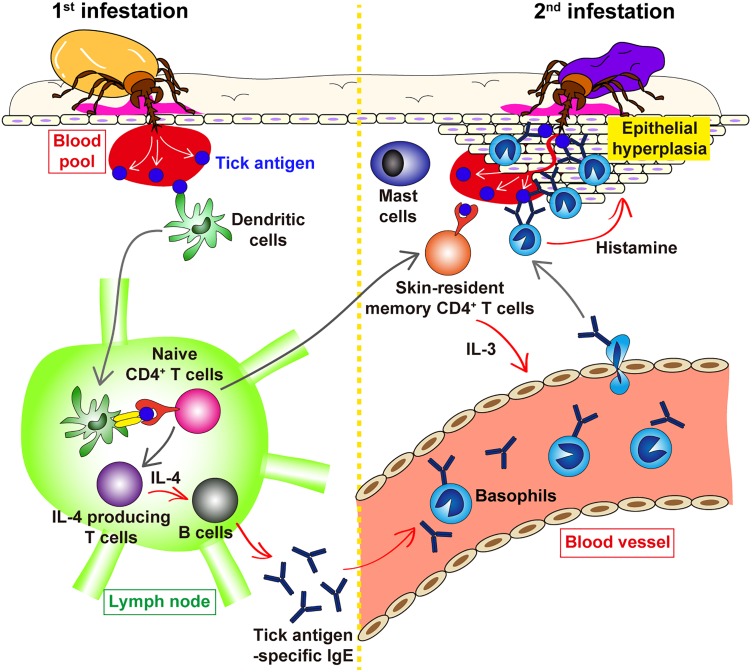
Schematic view of the proposed mechanism underlying ATR. In the 1^st^ tick infestation (left panel), dendritic cells in the skin take up tick saliva antigens and move to the draining lymph node where they present tick antigens to naive CD4^+^ T cells, leading to the generation of IL-4-producing T cells. T cell-derived IL-4 stimulates B cells to produce tick antigen-specific IgE that in turn circulates in the peripheral blood and bind to the surface of blood-circulating basophils via FcεRI. Some of tick antigen-specific CD4^+^ T cells generated in the lymph node migrate into the skin throughout the body and are retained as skin-resident, memory CD4^+^ T cells. In the 2^nd^ tick infestation (right panel), such skin-resident, memory CD4^+^ T cells are stimulated with tick antigens to produce IL-3 that in turn promotes the recruitment of IgE-armed basophils from the peripheral blood to the tick-feeding site. IgE-armed basophils are activated with tick antigens to release histamine that acts on keratinocyte, resulting in epidermal hyperplasia that may interfere with tick attachment or blood feeding in the skin, and hence contribute to ATR. The role of skin mast cells in ATR remains elusive.

It was shown in guinea pigs that transfer of serum from previously infested animals conferred ATR on naive animals (Wikel and Allen, 1976; Brown and Askenase, 1981; Askenase et al., 1982). Similarly, in mice, transfer of serum from tick-infested but not un-infested mice conferred ATR on naive mice (Matsuda et al., 1990), suggesting the involvement of tick-specific antibodies in ATR. Of note, the heat treatment of the serum at 56°C for 2 h abolished the ATR transfer activity (Matsuda et al., 1990), indicating that antibodies of IgE isotype contribute to the manifestation of ATR. Consistent with this observation, we demonstrated that both antibody-deficient μMT mice and *Fcer1g*^−/−^ mice, that lack the expression of high affinity IgE receptor FcεRI, failed to show ATR (Wada et al., 2010). This suggested the following scenario (Figure 2). The 1^st^ infestation triggers the production of IgE against tick saliva antigens, and basophils and mast cells bind IgE on the cell surface via FcεRI. In the 2^nd^ infestation, tick saliva antigens delivered into the tick-feeding site bind to IgE on these cells, leading to the cross-linking of FcεRI and hence activation of these cells that may contribute to ATR.

Intriguingly, mast cell-deficient *Kit^W-sh/W-sh^* C57BL/6 mice reconstituted with mast cells derived from *Fcer1g*^−/−^ mice could manifest ATR as did mice reconstituted with wild-type mast cells (Wada et al., 2010), indicating that FcεRI on mast cells is dispensable for IgE-mediated ATR. In contrast, adoptive transfer of basophils isolated from previously infested wild-type, but not *Fcer1g*^−/−^, mice conferred ATR on naive mice (Wada et al., 2010). These results suggested that basophils rather than mast cells play a critical role in IgE-dependent ATR through FcεRI-mediated activation, even though both types of cells contribute to ATR.

Ticks inject a plethora of substances, including proteins, into the host during feeding (Wikel, 2013). However, it remains ill-defined which components among tick saliva injected are the major targets of IgE that is involved in ATR, even though a series of tick saliva antigens recognized by sera from tick-infested animals and humans have been identified (Brown et al., 1984; Brown, 1988; Mayoral et al., 2004). It was demonstrated that infestation with *A. americanum* can induce a strong IgE response to tick saliva antigens including the carbohydrate α-gal, which is also present in red meats such as beef and pork. The production of such anti-α-gal IgE in tick-infested people can lead to anaphylaxis after ingestion of red meats (Platts-Mills and Commins, 2013; Steinke et al., 2015). It remains to be investigated whether anti-α-gal IgE is involved in ATR.

Molecular characterization of tick salivary components has demonstrated that different members among the same multi-gene family are expressed at distinct time points during tick feeding (Karim and Ribeiro, 2015). For example, two cystatin genes from *Ixodes scapularis* change their expression reciprocally during feeding (Kotsyfakis et al., 2007; Karim and Ribeiro, 2015). Such antigenic variation or sialome switch during tick feeding is considered as a possible mechanism by which ticks avoid host immune responses. It remains to be determined whether such variation can affect the production of anti-tick IgE and hence IgE-mediated ATR, and whether IgE raised against one family member is cross-reactive to other members of the same family.

Host-derived IgG molecules containing blood meal pass through the midgut barrier of *Rhipicephalus appendiculatus* into the hemolymph and are excreted via the saliva back into the host during feeding. IgG binding proteins detected in the tick hemolymph and salivary glands are thought to contribute to this excretion of IgG, as a strategy by which ticks evade the damage caused by host antibodies (Wang and Nuttall, 1999). IGBP-MA, a member of IgG binding proteins has been shown to bind to IgE (Wang and Nuttall, 2013). Further studies are needed to examine whether such IgG binding proteins can interfere with IgE-mediated ATR in the host and whether the host raises antibodies against them to neutralize their activity.

## Basophil-Derived Histamine is an Important Effector Molecule in ATR

Biologically active molecules, such as histamine and proteases, stored in the secretary granules in basophils and mast cells have been implicated as effectors of ATR. It was reported in cattle that the tick resistance is correlated with hypersensitivity to tick antigens and the amount of histamine at the tick-feeding site (Willadsen et al., 1979). Moreover, administration of antihistamine in cattle resulted in higher tick numbers (Tatchell and Bennett, 1969) whereas the injection of histamine into the cattle skin promoted tick detachment (Kemp and Bourne, 1980). Similar observations were reported in guinea pigs (Wikel, 1982), suggesting the possible involvement of histamine to ATR. However, the cellular source of histamine responsible for ATR and the mechanism underlying histamine-mediated ATR remained ill-defined.

We have recently addressed these questions by analyzing C57BL/6 mice infested with *H. longicornis*, in that both basophils and mast cells contribute to ATR (Wada et al., 2010). Treatment of mice with histamine H1 antagonist during the 2^nd^ infestation abolished ATR (Tabakawa et al., 2018). Consistent with this, mice deficient for histamine production due to the lack of histidine decarboxylase (HDC) failed to show ATR (Tabakawa et al., 2018). Moreover, repeated injection of histamine or histamine H1 receptor agonist beneath the tick-infested site during the 1^st^ infestation inhibited the tick feeding in wild-type mice (Tabakawa et al., 2018). These observations illustrated the important role of the histamine-histamine H1 receptor axis in the manifestation of ATR in mice, consistent with previous studies in guinea pigs and cattle (Tatchell and Bennett, 1969; Willadsen et al., 1979; Wikel, 1982).

Both basophils and mast cells are well-known producers of histamine, and therefore supposed to contribute to ATR through histamine release. Unexpectedly, however, adoptive transfer of histamine-deficient mast cells reconstituted ATR in mast cell-deficient *Kit^W-sh/W-sh^* C57BL/6 mice as did that of wild-type mast cells (Tabakawa et al., 2018), indicating that mast cell-derived histamine is dispensable for ATR. In contrast, adoptive transfer of wild-type but not histamine-deficient basophils conferred ATR on basophil-depleted *Mcpt8*^DTR^ mice (Tabakawa et al., 2018), demonstrating the crucial role of basophil-derived histamine in the manifestation of ATR.

Intravital imaging analysis of cells at the 2^nd^ tick feeding site demonstrated that basophils make a cluster within the epidermis and surround a tick mouthpart. In contrast, mast cells are mostly scattered in the dermis rather than epidermis and localized more distantly from the tick mouthpart (Tabakawa et al., 2018). It is well known that histamine has a short half-life. Therefore, basophil-derived histamine may be much more effective than mast cell-derived in the manifestation of ATR, considering the fact that higher numbers of basophils are localized closer to a tick mouthpart, compared to mast cells.

Previous studies reported that histamine promotes itching and grooming response in the skin, resulting in removal of ticks in host animals (Koudstaal et al., 1978). In the mouse mode of tick infestation, ticks are placed inside of a small tube attached to the skin. Therefore, the effect of host grooming on tick feeding is minimized, implying other mechanisms underlying histamine-mediated ATR. Mice deficient for histamine H1 receptor failed to manifest ATR (Tabakawa et al., 2018), indicating that histamine acts on host cells rather than ticks. We detected the thickening of the epidermis and the formation of basophil cluster within the thickened epidermis at the 2^nd^ but not 1^st^ tick-feeding site in mice (Tabakawa et al., 2018) as reported previously in guinea pigs (Trager, 1939; Allen, 1973). This epidermal hyperplasia was absent in histamine-deficient or basophil-deficient mice (Tabakawa et al., 2018), suggesting that basophil-derived histamine is involved in epidermal hyperplasia. Considering that keratinocytes express functional H1 receptor (Ohsawa and Hirasawa, 2014) and that histamine promotes the proliferation of keratinocytes (Maurer et al., 1997; Albrecht and Dittrich, 2015), histamine released from basophil localized in the epidermis perhaps induces the thickening of the epidermis that may interfere with tick attachment or blood-sucking in the skin during the 2^nd^ infestation (Figure 2).

Histamine-binding proteins (HBPs) have been identified in tick saliva (Paesen et al., 1999; Sangamnatdej et al., 2002; Mans et al., 2008). They show high-affinity binding to histamine and can efficiently compete for histamine with its native receptor. Thus, they may interfere with histamine-induced host responses at tick feeding sits, including itching and grooming. However, it remains to be determined whether tick HBPs can give any impact on histamine-mediated ATR in the host and whether the host raises antibodies against them to neutralize their activity. Mast cells and basophils are the major source of histamine at tick feeding sites. Basophils accumulate at tick feeding sites during the 1^st^ but not 2^nd^ infestation while mast cells always reside there. Given that higher numbers of basophils are localized closer to a tick mouthpart, compared to mast cells, during the 2^nd^ infestation (Tabakawa et al., 2018), the concentration of histamine near tick mouthparts should be much higher during the 2^nd^ infestation compared to the 1^st^ infestation. Therefore, one may assume that HBPs might be less effective in sequestering histamine at the 2^nd^ tick-feeding site in which ATR is executed. The influence of HBPs on histamine-mediated ATR could be explored by generating HBP-deficient ticks in future studies.

It has been reported that *H. longicornis*, *Dermacentor andersoni*, and *Boophilus microplus* larval ticks are highly reactive to histamine in the induction of tick resistance while *A. americanum* and *Ixodes holocyclus* ticks are less responsive to histamine (Bagnall, 1975; Kemp and Bourne, 1980; Wikel, 1982; Brown and Askenase, 1985). The former tick species have shorter mouthparts than the latter (Suppan et al., 2017), suggesting the possibility that histamine-induced thickening of the epidermis prevents the former’s but not the latter’s mouthparts from penetrating into the dermis in order to form blood pools. This may explain the differential responsiveness to histamine among tick species in terms of ATR induction. Alternatively, but not mutually exclusively, it is possible that the presence or absence (or differential amounts) of HBPs in different tick species is correlated in part with differential reactivity to histamine in the induction of tick resistance.

## Skin-Resident Memory Cd4^+^ T Cells Are Responsible for Basophil Recruitment to the 2^nd^ Tick-Feeding Site

Basophils circulate in the peripheral blood under homeostatic conditions, and they infiltrate the skin at the tick-feeding site during the 2^nd^ but not 1^st^ infestation. Importantly, the recruitment of basophils can be observed in previously uninfested skin, far from the 1^st^ infestation site, of sensitized animals, implicating that the 1^st^ tick infestation may induce systemic alteration in the skin throughout the body, so that basophils can readily infiltrate the tick re-infestation site anywhere in the body. We have recently demonstrated in mice that skin CD4^+^ memory T cells play an important role in basophil recruitment to the 2^nd^ tick-feeding site, leading to ATR (Ohta et al., 2017). Tick antigen-specific CD4^+^ effector T cells are generated during the 1^st^ tick infestation and distributed to the skin all over the body, and some of them are retained as skin-resident memory T cells (Figure 2). In the 2^nd^ tick infestation, tick saliva antigens delivered into the skin stimulate these memory T cells present in the skin to produce IL-3 that is required for basophil recruitment to the 2^nd^ tick-feeding site (Ohta et al., 2017). Even though the exact mechanism underlying IL-3-mediated basophil recruitment remains to be clarified, IL-3 might promote basophil adhesion to endothelium (Bochner et al., 1990; Korpelainen et al., 1996; Lim et al., 2006), leading to transendothelial migration of basophils and their accumulation in the skin.

## An Unsolved Issue: the Role of Mast Cells in ATR

As described above, mast cells contribute to ATR in mice infested with *H. longicornis* (Matsuda et al., 1985, 1987, 1990) whereas they are dispensable for ATR in *D. variabilis*-infested mice (denHollander and Allen, 1985; Steeves and Allen, 1991). As far as we are aware, the involvement of mast cells to ATR has not yet been documented in other animal species. In the case of mice infested with *H. longicornis*, histamine derived from basophils but not mast cells is essential for the manifestation of ATR (Tabakawa et al., 2018), even though both basophils and mast cells are involved in ATR (Wada et al., 2010). The deficiency of either basophils or mast cells almost completely abolishes ATR (Wada et al., 2010), suggesting that the role of these cells may not be additive. Of note, the number of basophils accumulating at the 2^nd^ tick-feeding site is comparable between mast cell-sufficient and -deficient mice (Wada et al., 2010), indicating that mast cells are not prerequisite for basophil recruitment. Nevertheless, closer examination with intravital imaging revealed that basophils accumulating at the 2^nd^ tick-feeding site are more motile and less-clustered around a tick mouthpart in mast cell-deficient mice than in mast cell-sufficient mice (Tabakawa et al., 2018). Therefore, one may assume that mast cells may contribute to ATR by directly or indirectly regulating basophil behavior. Further studies are needed for elucidating how mast cells contribute to the manifestation of ATR.

## Conclusion

Recent development of a series of analytical tools in laboratory animals has advanced our understanding of the cellular and molecular mechanism underlying ATR. In several animal species, basophil accumulation is observed at the tick re-infestation site (Figure 1), and basophil depletion abolishes ATR in guinea pigs and mice, demonstrating the crucial role of basophils in the manifestation of ATR. The 1^st^ tick infestation triggers the production of IgE against tick saliva antigens. In the 2^nd^ infestation, IgE-armed basophils are recruited to the tick-feeding site and stimulated by tick saliva antigens to release histamine that functions as a key effector in ATR, probably through promotion of the epidermal hyperplasia that in turn interferes with tick attachment or blood feeding in the skin (Figure 2). Further studies on the detailed mechanism underlying ATR, including the role of mast cells, may help develop the strategy to prevent tick infestation and tick-borne diseases.

## Author Contributions

HK has substantially contributed to the literature review and drafted the manuscript. SY contributed to the discussion and drafting, and editing of the manuscript. YT, TO, and TW reviewed and edited the manuscript.

## Conflict of Interest Statement

The authors declare that the research was conducted in the absence of any commercial or financial relationships that could be construed as a potential conflict of interest.
